# Development of Floating Tablets of Metformin HCl by Thermoplastic Granulation. Part II: *In Vitro* Evaluation of the Combined Effect of Acacia Gum/HPMC on Biopharmaceutical Performances

**DOI:** 10.34172/apb.2020.048

**Published:** 2020-05-11

**Authors:** Mohamed Djebbar, Nacéra Chaffai, Fatiha Bouchal

**Affiliations:** ^1^Galenic Pharmacy Laboratory, Pharmacy Department Medicine Faculty, Badji Mokhtar University, P.O. Box 204 Route Zaafrania, Annaba, Algeria.; ^2^Pharmaceutical Laboratory, Department of Engineering Process, Faculty of Technology, Abderrahmane-Mira University, Bejaia, Algeria.

**Keywords:** Acacia gum, Acacia gum/HPMC Combination, Floating tablets, Metformin HCl, Stearic acid, Thermoplastic granulation

## Abstract

***Purpose:*** The aim of this study was to evaluate the combined effect, acacia gum(AG)/ hydroxypropylmethylcellulose (HPMC), on biopharmaceutical performances of floating tablets of metformin hydrochloride (MTH) prepared by thermoplastic granulation using stearic acid.

***Methods:*** We have prepared the matrixes using AG/HPMC as a polymer combination. This combination of polymers which represents 15% of the total mass of tablet was used at various ratios 3:1, 1:1, 1:3, with two viscosity grade of HPMC (k15M and k100M). The developed matrixes have been evaluated for their pharmacotechnical and biopharmaceutical properties.

***Results:*** In addition to the satisfactory physical characteristics of matrixes, it was revealed that the Fc3 and Fc6 formulations with AG/HPMC k15M and AG/HPMC k100M respectively, at ratio, 1:3 were considered the most performance. These formulations have shown swelling, fast flotation, 360 and 480 seconds respectively, and remained floating on the surface of the medium for more than 24 hours, with the matrix integrity, while F1, containing only AG, did not show swelling and did not float. In addition, extended*in vitro* release (>8 hours) with decreased dissolved MTH rates was demonstrated for Fc3 and Fc6 matrixes, 95% and 91% respectively, compared to F1 where MTH dissolution was complete after 2 hours. The drug release from the highest-performance matrixes (Fc3 and Fc6) was found to follow Korsmeyer-Peppas’s model. The mechanism drug release was controlled by diffusion and erosion.

***Conclusion:*** The AG/HPMC combination was suitable as a polymer matrix to improve the *in vitro* biopharmaceutical properties of MTH compared to AG.

## Introduction

Metformin hydrochloride (MTH) is a drug with a short half-life and a narrow absorption window in the upper gastrointestinal tract. In addition, this drug belongs to the biopharmaceutical classification system, BCS III. In this case, the development of floating tablets is a real alternative to extend the gastric residence time of MTH. Indeed, this makes it possible to sustain and prolong its release and, consequently, to improve its bioavailability in the upper part of the small intestine. In this perspective, several manufacturing techniques allow the design of such systems. Djebbar et al^1^ have demonstrated that melt granulation of MTH using stearic acid with HPMC k15M or HPMC k100M, according to an effervescent approach (NaHCO_3_), makes it possible to prepare of floating tablets. The developed floating matrices have showed fast flotation, total flotation time of more than 24 hours, matrix integrity and drug release extended (>8 hours) with significantly decreased drug dissolution rate (92% and 80%). However, in the same study, it was noted that among the studied hydrophilic polymers, the natural polysaccharide, acacia gum (AG), did not give any effective result. This is why in this work; we proposed to improve the biopharmaceutical performances of this polymer. In this perspective and according to the literature, the combination of hydrophilic polymers seems to be an interesting approach. Several studies aiming to develop sustained-release MTH floating tablets, have used this strategy based on the combination of hydrophilic polymers.

Roy et al^2^ have developed a matrix-controlled drug delivery system of metformin HCl by wet granulation using various grades of hydrophilic polymers, hydroxypropylmethylcellulose (HPMC k15M, HPMC k100M and HPMC k200M) and polyacrylate polymers (Eudragit RL100 and Eudragit RS100). It was shown that various grades of HPMC at suitable concentration combined with polyacrylate polymers could be used effectively to modify the release rates. The formulation F6 with polymer combination (26%HPMC k200M-Eudragit RS100) could sustain the drug release for 12 hours. In the same perspective, Nha et al^3^ have developed new metformin-based sustained-release floating systems by wet granulation using polymer combinations for the prolonged release and absorption. It was concluded from the evaluation of these systems that the combination of HPMC k15M and HPMC k100M with the ratio of 90:260 w/w and the amount of NaHCO_3_ and citric acid at 65 mg and 13 mg, respectively, benefits the floating sustained release tablet formulation with a lower floating lag time to 1 minute. The same observations were reported in the work of Bahri-Najafi et al^4^ with the aim of developing a floating drug delivery system based on MTH. It has been exhibited that F7 formulation (300HPMC k4M–50PVP) is a promising system revealing excellent floating properties and sustained drug release characteristics (MDT=5.26h and DE_12h_49.88%). These conclusions are supported by Senjoti et al.^5^ who have developed sustained-release floating tablets of metformin HCl based on swelling and effervescence. The developed tablets contained an effervescent agent (NaHC0_3_), a polymer combination (HPMC-PEO) and a swelling enhancer (SSG). The study has showed that flotation properties and drug release profile of developed tablets were influenced by the amount of HPMC, PEO, NaHCO_3_ and SSG. The optimized tablets of the study have showed remained floating for more than 24 hours with a floating lag time of less than 4 minutes and sustained-release of 12 hours. In addition, accelerated stability study (40°C and 75% RH) revealed that tablets from optimized formulation were stable for three months without any major changes in assay, dissolution profile, floating lag time, and other physical properties. Finally, the authors propose the HPMC-PEO, polymer combination, for being exploited in the future as a swelling polymer for floating systems to effectively increase the residence time of the drug in the stomach. Similarly, Patel et al^6^ have developed by direct compression technique effervescent floating tablets of metformin using a combination of polymers. The study revealed that the prepared formulation F2 using kappa carrageenan in combination with HPMC k15M is best in terms of showing excellent floating properties, extended adhesion periods and sustained drug release characteristics.

The polymer combination approach for designing floating tablets appears to provide promoter systems exhibiting excellent floating properties and satisfactory extended release characteristics of MTH. However, it should be noted that these systems are often prepared either by wet granulation or direct compression. The development of floating MTH tablets by thermoplastic granulation in the presence of a natural polysaccharide, AG combined with HPMC, was not investigated. With this in mind and in order to improve the performance of MTH floating tablets based on AG,^1^ in the present investigation, it is proposed to prepare matrix-tablets of metformin based on AG in combination with HPMC, using a simple solvent-free manufacturing process, the thermoplastic granulation. Therefore, the aim of this work was to evaluate the combined effect of AG–HPMC on the biopharmaceutical properties of MTH: *in vitro* flotation, swelling kinetics and *in vitro* drug release kinetics of MTH. The study has carried out on AG and HPMC at two viscosity grades, HPMC k15M and HPMC k100M. The AG–HPMC polymers combination, whose total polymer concentration represents 15% of the tablet’s mass, was evaluated in three ratios: 3:1, 1:1 and 1:3.

## Materials and Methods

### Chemicals

Metformin hydrochloride (MTH) (Shangai Pharmaceutical, China), Stearic acid (SA) (Transmare Antwerpen NV, Belgium), HPMC: HPMC k15M (Colorcon^®^, Royaume-Uni), HPMC k100M (ASHLAND, Germany), AG (AxoPharma, Belgium), sodium bicarbonate (SB) (Sigma-Aldrich, Germany), microcrystalline cellulose pH 102 (CMC) (JRS Pharma, France), Talc (Guangxi Longguang Talc) and magnesium stearate (Mg Stearate) (Development Co., Ltd., China and Anhui Sunhere Pharmaceutical Excipients Co., Ltd., China).^1^

### Formulation of MTH floating tablets

The combination of two hydrophilic polymers (AG–HPMC) in three ratios of 3:1, 1:1 and 1:3 was used to prepare the MTH floating tablets. Six formulas were developed with the combination, AG–HPMC, Fc1 to Fc6. It should also be noted that HPMC was used at two viscosity grades (k15M and k100M). The formulas, Fc1 to Fc3 contain AG–HPMC k15M, and Fc4 to Fc6 are based on AG–HPMC k100M. In Table 1, we have mentioned these formulas as well as those from a previous study where the same polymers were used separately, F1 (AG), F2 (HPMC k15M) and F3 (HPMC k100M).^1^

**Table 1:** Formulation of MTH floating tablets. Combined effect (AG–HPMC)

**Formulation**	**Composition (mg)**								
	**MTH**	**Stearic Acid**	**NaHCO**_3_	**HPMC k15M**	**HPMC k100M**	**Acacia Gum**	**CMC**	**Talc**	**Mg Stearate**
F1	250	100	50	-	-	75	Qs 500	7.5	2.5
F2	250	100	50	75		-	Qs 500	7.5	2.5
F3	250	100	50	-	75	-	Qs 500	7.5	2.5
Fc1	250	100	50	18.75	-	56.25	Qs 500	7.5	2.5
Fc2	250	100	50	37.50	-	37.50	Qs 500	7.5	2.5
Fc3	250	100	50	56.25	-	18.75	Qs 500	7.5	2.5
Fc4	250	100	50	-	18.75	56.25	Qs 500	7.5	2.5
Fc5	250	100	50	-	37.50	37.50	Qs 500	7.5	2.5
Fc6	250	100	50	-	56.25	18.75	Qs 500	7.5	2.5

Fc: Formulation using combination of polymers; F: Formulation based on one polymer

### Manufacture of MTH floating tablets

The MTH floating tablets were prepared using a simple process adopted in a previous study,^1^ the melt granulation. In a first step, weighing the drug and previously screened pharmaceutical excipients (sieve; 800 µm), melting of stearic acid at 58°C and dry mixing of MTH, CMC, NaHCO_3_ and combined polymers (AG–HPMC). In a 2^nd^ step, adding this mixture to the molten stearic acid, mixing and cooling the resulting mass. Finally, after solidification of this mass, calibration (sieve; 1000 µm), lubrication of the obtained granules (1.5% talc and 0.5% Mg stearate), then compression using an alternative press (Frogerais Type A 307), equipped with a double set of flat punches and a 12 mm diameter die. Each tablet of 12 mm diameter contains 250 mg of MTH and weights 500 mg.

### Pharmacotechnical study

#### Evaluation of granules

The prepared granules of MTH were evaluated for angle of repose, bulk and tapped densities, Carr’s index and Hausner’s ratio.

#### Physical characteristics of tablets

All the formulated tablets were evaluated for drug content, weight variation, hardness, friability and thickness.^7,8^

### In vitro buoyancy

The *in vitro* buoyancy was performed according to the method described by Jiménez-Castellanos et al.^9^ The time required for the tablet to rise to the surface and float, floating lag time (FLT) expressed of seconds (s) and the total floating time (TFT) expressed of hours (h) were determined. The results represent the average of 3 measures.

### Swelling index

The swelling index (SI) percent was calculated by using the following equation:

$$Swelling\;index(\%)=\frac{Wt-Wo}{Wo}\times 100$$

Where, Wt = Weight of tablet at time t, W_0_ = Initial weight of tablet. The swelling characteristics of floating tablets were expressed as % in terms of weight gain as a function of time. The results are the average of three measures.

### In vitro drug release kinetics and mechanism

For evaluating the effect of AG–HPMC, combination on the MTH release, the *in vitro* dissolution study was performed using the USP II paddle apparatus (Erweka DT 80), under the same operating conditions as those adopted in a previous study.^1^ The samples (5 mL) were collected from each test at 20 minutes, 40, 60, 90, 2 hours, then, each hour until 8 hours. The removed samples were filtered (filter; 0.45 µm), then analyzed with a UV-Vis spectrophotometer for MTH at 233 nm (Biochrom WPA Lightwave II). The drug release rate was calculated using calibration curve equation (y=0.0165x).^1^ The results represent the average of six measures (Mean ± CV). The mechanism of release was determined by fitting the *in vitro* release data to the various kinetic equations: Zero order, First order, Higuchi and model Korsmeyer-Peppas.^10-21^ For the high-performance formulations, the coefficient of determination (R^2^) and Akaike information criteria (AIC)^22,23^ of the dissolution profile corresponding to each model were determined. The highest R^2^ and the lowest AIC of a model nominated it as the best fitting model. The times required for 25%, 50% and 80% drug release (T_25%_, T_50%_ and T_80%_) from these formulas were determined according to the best fit model. The mean dissolution times (MDT) were also calculated.^11,24^ Finally, the diffusion exponent, “n” was calculated for characterizing the different release mechanisms of MTH from matrix. Fickian diffusion (n=0.45), non-Fickian diffusion (0.45<n<0.89), case II transport (n=0.89) and super case-II transport (n>0.89).^25^

*Zero order:* Q_t_ = Q_0_ + K_0_t

*First order*:

$$LogQt=LogQ0-\frac{Kt}{2,303}$$

*Higuchi model:* Q_t_= K_H_ t ^½^

$$Korsmeyer\;and\;Peppas\;\mathrm{mod}el:Log(\frac{Mt}{M\infty })=\log\;k+n\;\log\;t$$

T_25%_ = (25/k)^1/n^; T_50%_ = (50/k)^1/n^; T_80%_ = (80/k)^1/n^;$MDT=\frac{\sum_{i}ti\Delta Mi}{\sum_{i}\Delta Mi}$

AIC = n × ln (WRSS) + 2p (n is the number of dissolution data points, p is the number of parameter of the model, WSSR is the weighed sum square residual).

### FTIR spectroscopy

The characterization of MTH in the prepared matrixes was performed using FTIR spectral analysis (Shimadzu IR Affinity 1SCE) for any change in its physical state or any chemical interaction if any between the drug and the pharmaceutical excipients, particularly the melting agent. Samples of pure drug (MTH), stearic acid (SA), AG, HPMC, sodium bicarbonate (SB) and high-performance floating tablets were scanned in the spectral region of 4000 to 750 cm^-1^ with a resolution of 4 cm^-1^.

### Statistical processing 

The statistical analysis of the drug release kinetics data was carried out with GraphPad Prism 7 software to evaluate the MTH release profiles from developed floating systems. The evaluation was based on comparison between dissolution profiles according to the ratio of the combination (AG–HPMC) and the viscosity grade of the HPMC used in the polymers combination. We also compared the dissolution profiles of the matrices (Fc), developed with AG-HPMC combination, with those of the matrices (F), containing the same polymers separately. A one-factor variance analysis (ANOVA) was applied. The differences were considered significant at p < 0.05 with a 95% confidence interval.

## Results and Discussion

### Pharmacotechnical study

#### Evaluation of granules

The pre-compression parameters of the studied formulas are recorded in Table 2. In addition to formulations containing AG and HPMC separately (F1 to F3), for which good rheological properties have been demonstrated,^1^ all the formulations of this study have exhibited good flowability and compressibility properties regardless of the ratio of AG-HPMC, combination. It was noted: angle of repose in the range 33.66 to 35.37°, V_10_ - V_500_ between 11 and 16 mL, Carr’s index between 13.20 and 15.73% and Hausner’s ratio between 1.15 and 1.18 (Table 2).

**Table 2:** Evaluation of flow properties. Combined effect (AG–HPMC)

**Formulation**	**Angle of repose (°)**	**Bulk density (g/mL)**	**Tapped density (g/mL)**	**V**_10_ **- V**_500_ **(mL)**	**Carr’s index (%)**	**Hausner’s ratio**
F1	32.61	0.595	0.689	11	13.69	1.15
F2	33.50	0.492	0.546	12	10.34	1.11
F3	34.91	0.470	0.529	13	11.26	1.12
Fc1	34.79	0.520	0.609	11	14.58	1.17
Fc2	33.66	0.510	0.602	13	15.30	1.18
Fc3	35.09	0.471	0.543	16	13.20	1.15
Fc4	35.37	0.546	0.645	12	15.30	1.18
Fc5	35.37	0.534	0.629	12	14.97	1.17
Fc6	34.60	0.507	0.602	15	15.73	1.18

#### Physical characteristics of tablets

The tablets, Fc (AG–HPMC) obtained by melt granulation with stearic acid have manifested satisfactory and reproducible physical characteristics as shown in Table 3. The average content of metformin HCl is between 98.78 and 101.18%, the mass uniformity is within the prescribed limits by European pharmacopoeia^8^; 500 ± 25 mg, the hardness varies from 4.87 ± 0.54 to 5.19 ± 0.31 kg/cm^2^. The thickness and percentage of friability are between 3.63 ± 0.06 to 3.71 ± 0.02 mm and 0.32 to 0.87% respectively.

**Table 3:** Post-compression parameters. Combined effect (AG–HPMC)

**Formulation**	**Average Drug content ( %)** ^b^	**Weight variation (mg ± SD)** ^a^	**Hardness (kg/cm** ^ 2 ^ **± SD)** ^b^	**Thickness (mm ± SD)** ^b^	**Friability (%w/ w)** ^b^
F1	97.63	494.5±0.87	5.10±0.51	3.43±0.03	0.88
F2	98.19	509.8±0.96	5.31±0.13	3.65±0.04	0.80
F3	101.79	506.65±1.44	5.23±0.16	3.73±0.08	0.73
Fc1	101.18	507.55±1.11	5.08±0.36	3.68±0.03	0.43
Fc2	98.78	503.3±0.91	5.07±0.20	3.63±0.06	0.46
Fc3	98.82	500.1±0.88	5.19±0.31	3.67±0.03	0.32
Fc4	100.60	502.55±0.95	5.19±0.24	3.68±0.03	0.50
Fc5	98.80	498.9±0.98	4.87±0.54	3.71±0.02	0.82
Fc6	99.40	502.85±1.01	5.18±0.35	3.67±0.05	0.87

^a^n= 20, ^b^n= 10, SD: Standard deviation.

### In vitro buoyancy

The evaluation of the *in vitro* buoyancy study reported in Table 4 reveal that the buoyancy properties of metformin HCl tablets are dependent of the ratio of the combination (AG–HPMC) and of the HPMC viscosity grade. From the buoyancy evaluation, it emerges: formulations Fc1 and Fc4, AG–HPMC (3:1) containing HPMC k15M and HPMC k100M respectively, showing respective flotation times of 1560 and 1020 seconds have manifested signs of fragmentation and disintegration with the total flotation time of less than 6 hours. Fc2, AG–HPMC k15M (1:1) has shown a buoyancy time of 840 seconds, a disintegration of the pharmaceutical form and TFT< 8 hours. With the same ratio, 1:1 but, in the case of HPMC k100M, the Fc5 formula, with fast FLT, 480 seconds, and TFT >24 h has indicated surface erosion of the shape. Finally, the tablets Fc3 and Fc6, AG–HPMC (1:3) containing HPMC k15M and HPMC k100M respectively have indicated fast FLTs, 480 and 360 seconds respectively with the matrix integrity maintained and the total flotation time greater than 24 hours. It was concluded that compared to the AG used only (F1),^1^ the combined effect of the AG with the HPMC is beneficial. It results in a significant decrease in FLT. This observation is particularly pronounced in the case where the ratio is in favor of the concentration of the HPMC; AG–HPMC (1:3). Indeed, the HPMC at a sufficient concentration in the combination, AG–HPMC is able to form, following its hydration, a gelled barrier sufficiently resistant to the pressures generated by the release of carbon dioxide bubbles and the agitation of the medium. Also, we found that the improvement in buoyancy properties was more pronounced in the case of the high-viscosity grade HPMC. TFT>24 hours for Fc5 compared to TFT<8 hours for Fc2 and FLT=360 seconds for Fc6 compared to FLT=480 seconds for Fc3 (Table 4). The AG–HPMC (1:3), combination has proven to be the most performing. This combination has shown fast flotation, prolonged flotation and matrix integrity (24 hours).

**Table 4:** Evaluation of combined effect (AG–HPMC) on the *in vitro* buoyancy of metformin HCl tablets

**Formulation**	**FLT (s)** ^a^	**Matrix integrity**	**TFT (h)**
F1	> 3600	+++	> 24
F2	360±20	+++	˃ 24
F3	300±30	+++	˃ 24
Fc1	1560±30	Disintegration	< 6
Fc2	840±20	Disintegration	< 8
Fc3	480±15	+++	> 24
Fc4	1020±25	Disintegration	< 6
Fc5	480±10	Superficial erosion	> 24
Fc6	360±30	+++	> 24

^a^n= 3.

### Swelling index

**Figure 1:**
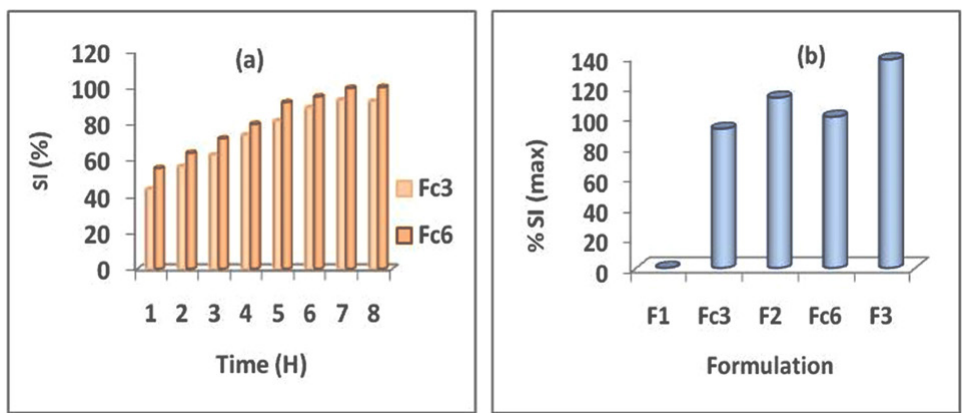
Swelling kinetics **(a)** Effect of HPMC viscosity grade in AG–HPMC combination at 1:3 ratio, Fc3-Fc6 **(b)** comparative study between SI max of MTH matrix-tablets based on AG–HPMC combination at ratio 1:3 and on polymer used alone (AG or HPMC): F1 (AG), Fc3-F2 (HPMC k15M) and Fc6-F3 (HPMC k100M).

Unlike the F1 matrixes containing only the AG which have not swell,^1^ the prepared floating tablets with the AG–HPMC, combination at the ratio 1:3, corresponding at Fc3 and Fc6 formulations have shown swelling (Figure 1a). Also, it was noted with these formulations an influence not negligible of the HPMC viscosity grade on the swelling kinetics. It was found the highest swelling indexes with the highest viscosity grade of HPMC (HPMC k100M). At the end of the swelling kinetics, it was noted a maximum swelling index equal to 100.07% for Fc6 compared to 92.18% for Fc3 (Figure 1a). Result in accordance with the observations of the buoyancy test which indicated fast buoyancy for these formulations.

In order to carry out a comparative study, the maximum swelling indexes (SI max) for all formulations that have shown swelling have reported in Figure 1b. It appears that despite the swelling manifested by floating systems containing AG combined with HPMC (Fc3 and Fc6), the maximum swelling indexes remain lower than those of tablets containing only HPMC. Figure 1b has shown a SI max of 92.18% for Fc3 (AG–HPMC k15M) compared to 112.72% for F2 (HPMC k15M) and of 100.07% for Fc6 (AG–HPMC k100M) compared to 138.11% for F3 (HPMC k100M).

### In vitro drug release

**Figure 2:**
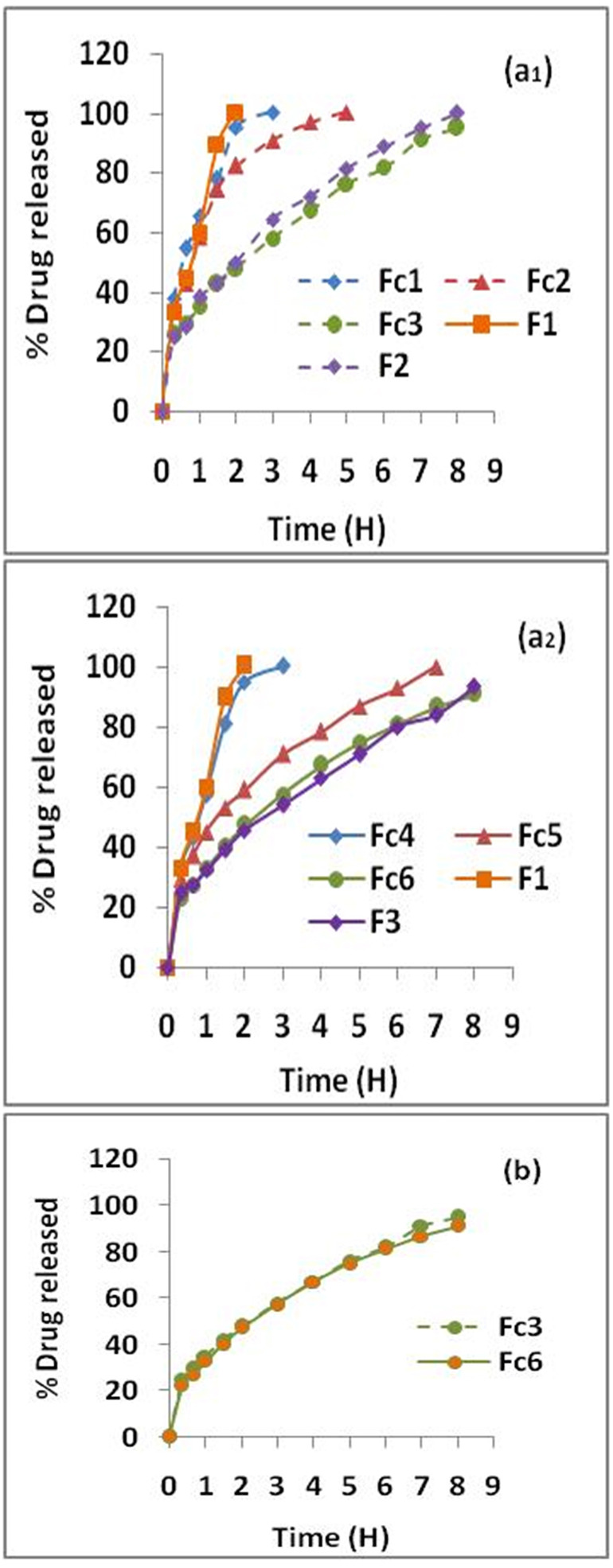
Dissolution kinetics of matrix-tablets **(a)** Effect of combination ratio (AG–HPMC) on MTH release: **(1)** AG–HPMC k15M (Fc1-Fc2-Fc3), **(2)** AG–HPMC k100M (Fc4-Fc5-Fc6) **(b)** Effect of HPMC viscosity grade of AG–HPMC combination on MTH release kinetics from high-performance floating tablets (Fc3 & Fc6).

On Figures 2a_1_ and 2a_2_ are depicted the dissolution profiles of MTH matrix-tablets containing AG combined with HPMC, Fc1 to Fc6 as well as the dissolution profiles of matrix-tablets prepared in the study realized by Djebbar et al,^1^ according to the same approach, melt granulation with stearic acid, in the presence of the same polymers, but used separately, F1 (AG), F2 (HPMC k 15M) and F3 (HPMC k100M). It is clear that the MTH release kinetics is slowed and prolonged from the formulations based on polymers combination (Fc), compared to floating matrixes containing only AG (F1) whose kinetics was complete after 2 hours.

The combined effect of AG–HPMC on the dissolution kinetics of metformin HCl was also evaluated according to the ratio of combination (AG–HPMC) (Figures 2a_1_ and 2a_2_) and the viscosity grade of HPMC (Figure 2b).

#### Effect of ratio (AG–HPMC)

*Case of HPMC k15M:* In general, dissolution profiles show significant differences (*P* <0.05). From Figure 2a_1_, it can be seen that the kinetics of MTH is a function of the *ratio* (AG–HPMC). It is slowed and extended with the increase in HPMC k15M concentration in the following order: Fc3˃Fc2≥Fc1=F1. The dissolution profiles for Fc1 and Fc2 tablets at the respective ratios (3:1) and (1:1) have indicated complete kinetics after 3 and 5 hours of testing respectively (Figure 2a_1_). This is due to the progressive disintegration of the floating tablets in the dissolution medium. Result in accordance with the conclusions of the buoyancy test which has indicated that these formulations reveal a disintegration of the pharmaceutical form and a TFT of less than 6 and 8 h respectively (Table 4). The AG–HPMC k15M combination at ratios (3:1); (1:1) therefore did not form a gelled barrier sufficiently resistant to CO_2_ released in contact with the H^+^ medium. However, compared to F1 (AG), which did not produce positive results,^1^ the combination of AG–HPMC k15M with the ratio (1:3) corresponding to the Fc3 formulation, with the highest concentration of HPMC, provided floating tablets whose dissolution kinetics of MTH is slowed and extended. Indeed, it was noted: 25.49% of dissolved drug after 20 minutes, 50% between 2 and 3 hours, 80% around 6 hours and 95.03% after 8 hours of testing (Figure 2a_1_). Finally, a significant difference between Fc3 and F1 was observed (*P* <0.05); the extended drug release follows the order: Fc3>F1. Result in accordance with the buoyancy and swelling tests. On the other hand, it should be noted that no significant difference was found between the dissolution profiles of the formulation containing AG–HPMC k15M, at the ratio (1:3), Fc3 and of the matrixes prepared with HPMC k 15M used alone, F2 (*P* ≥0,05).

#### Case of HPMC k100M

As for the HPMC k15M, dissolution profiles show significant differences (*P* <0.05). In the same way, the kinetics of MTH is a function of the ratio (AG–HPMC). It is slowed and extended with the increase in HPMC concentration in the combination (AG–HPMC k100M) and follows the following order: Fc6˃Fc5˃Fc4=F1 (Figure 2a_2_). The dissolution profile of the Fc4 formulation at the ratio (3:1) has indicated complete kinetics after 3 h of testing (Figure 2a_2_). As in the case of the HPMC k15M, this is due to the disintegration of the floating tablets in the dissolution medium clearly observed during the buoyancy test (Table 4). The combination, AG–HPMC k100M at the ratio (3:1) did not form a gel barrier sufficiently resistant to agitation and CO_2_ bubbles induced by the effervescence. The Fc5 formulation, at ratio 1:1 has shown a dissolution half-life time (t_50%_) of about 90 min. This relatively high release of MTH can be explained by surface erosion of the tablets upon contact with the test medium. The prepared tablets from this formulation have shown complete dissolution after 7 hours (Figure 2a_2_). However, compared to the formulation contained only AG, F1, which did not give conclusive results,^1^ the formulation Fc6, obtained with the combination (AG–HPMC k100M), at the ratio of 1:3 has provided floating tablets with significantly slower dissolution kinetics of MTH (>8 hours). Indeed, it was found: 22.83% of dissolved MTH after 20 minutes, 50% between 2 and 3 h, 80% around 6 h and 91.15% at the end of the test (8 hours) (Figure 2a_2_). A significant difference between Fc6 and F1 was reported (*P* <0.05). The extended drug release follows the order: Fc6>F1. Result in compliance with the buoyancy and swelling tests. Finally, no significant difference was noted between the dissolution profiles of the formulation containing AG–HPMC k100M, at the ratio (1:3), Fc6 and of the floating tablets based on HPMC k 100M used alone, F3 (*P* ≥0.05).

At the end of this evaluation, it was concluded that the combination (AG–HPMC) at the ratio 1:3 is the most performance. It was demonstrated a fast flotation time and a prolonged total flotation time. In addition, the release of MTH was slowed and extended with approximately 95% and 91% of dissolved drug after 8 hours for Fc3 and Fc6, respectively (Figures 2a_1_ and 2a_2_). The behavior of MTH floating tablets based on this combination (Fc3 and Fc6) could be explained by the properties of the HPMC which, at high concentration in the polymer combination and thanks to its hydration capacity, quickly forms a resistant gel barrier, trapping induced CO_2_ bubbles in contact with the H^+^ medium, thus promoting swelling, flotation of the form and control of drug release (extended release).

#### Effect of HPMC viscosity grade of the combination AG–HPMC

In addition to the extended drug release, Figure 2b has shown superposed MTH release profiles. This clearly illustrates that the dissolution kinetics of the Fc3 and Fc6 formulations are similar. No significant difference was noted (*P* ≥0.05). We can conclude that the viscosity grade of HPMC, combined with AG, has no effect on the release kinetics of MTH from floating matrixes prepared by melt granulation with stearic acid, unlike the observations made of the buoyancy and swelling tests which have indicated a non-negligible effect of the viscosity grade.

Finally, from the study of *in vitro* release, it emerges:

On the one hand, the improvement of MTH’s biopharmaceutical performances with the combination AG–HPMC, at 1:3 ratio (Fc3, Fc6/high performance), unlike the use of the natural polysaccharide, AG, alone (F1). Indeed, it was observed that the extended release follows the order following; Fc3>>>F1 and Fc6>>>F1.

On the second hand, despite the demonstrated similarity between the dissolution profiles of Fc3 with F2 and Fc6 with F3, it should be noted that the extended release of MTH is achieved with a low concentration of HPMC (11.25%) in the case of floating tablets Fc3 and Fc6, compared to 15% for tablets F2 and F3, containing HPMC alone.

#### Dissolution time of successive fractions

The combined effect of Acacia gum with HPMC (k15M or k100M) on dissolution times, T_25%_, T_50%_ and T_80%_ and the average dissolution time, MDT was also evaluated. The prepared tablets from the high-performance formulations (Fc3 and Fc6) have shown T_25%_, T_50%_, T_80%_, and MDT, delayed and therefore an extended MTH delivery. Indeed, it was registered respectively T_25%_, T_50%_ and T_80%_ of 0.5157 h-2.1401 h-5.6169 h and of 0.5576 h-2.2750 h-5.9019 h for Fc3 and Fc6 respectively. Also, MDT values equal to 2.6784 h and 2.5821 h for Fc3 and Fc6 respectively were registered. Finally, no significant difference between the dissolution times of the successive fractions of these formulations was observed. This explains the similarity of their dissolution kinetics, thus confirming that the HPMC viscosity grade has a negative effect on the release of MTH from developed floating matrixes in this study.

#### Kinetic modeling of in vitro dissolution data

The *in vitro* dissolution data of the floating tablets prepared from the performance formulations, Fc3 and Fc6 was analyzed with the different mathematical models of release kinetics. The values R^2^, k, AIC and n were recorded in Table 5. The *in vitro* dissolution profiles of Fc3 and Fc6 were satisfied the Korsmeyer-Peppas model with R^2^value of 0.998 and AIC values of −61.59 and −44.59 respectively. The drug release exponents “n”, for Fc3 and Fc6, are equal to 0.487 and 0.493 respectively. The dissolution of MTH from floating matrixes prepared by melt granulation with stearic acid in the presence of two combined polymers, AG and HPMC, at the ratio (1:3) has followed a non-Fickian transport mechanism (anomalous transport) (0.45<n<0.89). The MTH release was therefore controlled by diffusion and erosion (Table 5).

**Table 5:** Data modeling of dissolution kinetics of high-performance MTH floating tablets (Fc3, Fc6)

**Model**	**Parameters**	**Formulation**	
		**Fc3**	**Fc6**
Ordre Zéro	R^2^	0.983	0.966
	k	8.534	8.139
	AIC	35.61	40.48
Ordre Un	R^2^	0.958	0.992
	k	0.345	0.285
	AIC	−21.42	−41.18
Higuchi	R^2^	0.999	0.998
	k	33.74	32.71
	AIC	19.86	20.54
Korsmeyer-Peppas	R^2^	0.998	0.998
	k	34.51	33.34
	AIC	−61.59	−44.59
	n	0.487	0.493

#### FTIR spectroscopy

**Figure 3:**
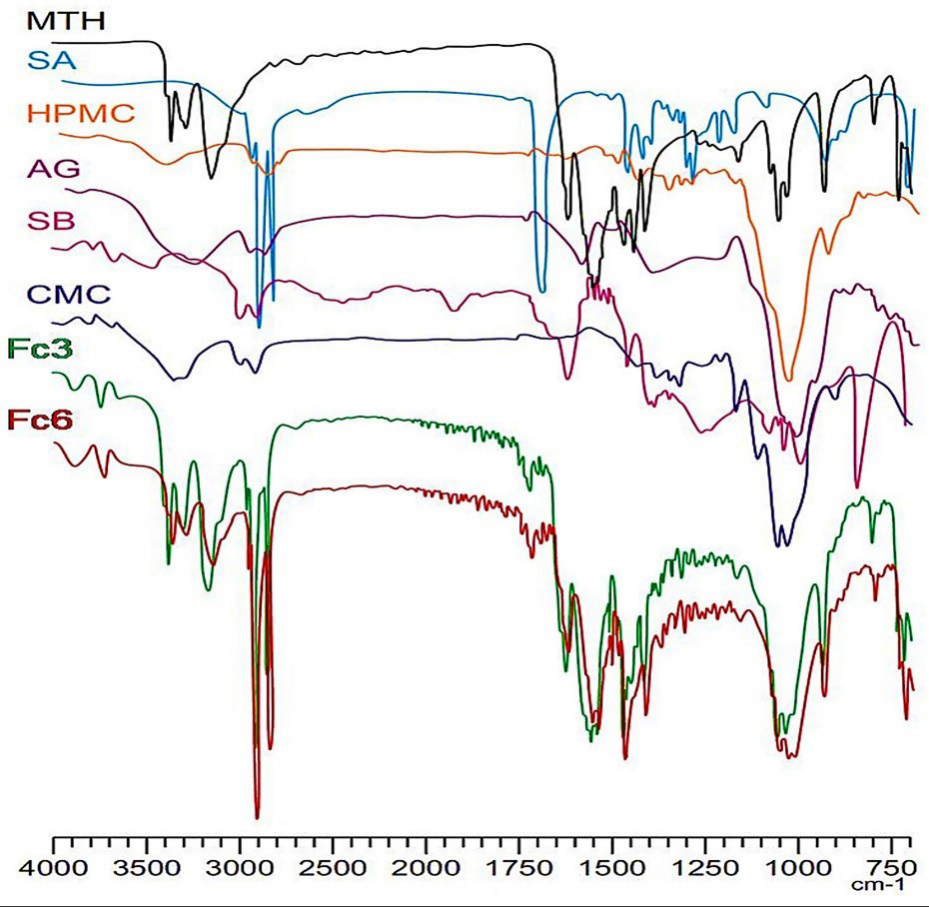
FT-IR spectra: Metformin HCl (MTH), Stearic acid (SA), Hydroxypropylmethylcellulose (HPMC), Acacia gum (AG), Sodium bicarbonate (SB), Carboxymethylcellulose (CMC) and high-performance floating matrixes (Fc3, Fc6).

The results of infrared spectroscopy confirmed the identity of metformin in the developed matrix-tablets by melt granulation with combined polymers at ratio 1:3 (Fc3 and Fc6). Also, the FTIR spectroscopy did not reveal any interaction between the drug and the excipients. This confirms the non-interaction between MTH and the melting agent (stearic acid). The infrared spectra of floating matrixes have clearly shown the characteristic absorption peaks of MTH; N-H in the region 3400 et 3100 cm^-1^; 3 High intensity absorption peaks around of, 3375, 3302 and 3173 cm^-1^, C=N; two intense absorption bands at 1632 cm^-1^ and 1576 cm^-1^, Aliphatic CH_3_; three absorption peaks around of, 1477, 1450 and 1410 cm^-1^, C-N of aliphatic diamines; low intensity bands in the region 1250-1050 cm^-1^ (Figure 3). These results are in accordance with the conclusions of the work carried out by Vaingankar and Amin^26^ where FTIR spectroscopic analysis of MTH extended release tablets prepared by melt granulation with stearic acid has suggested non-existence of chemical interaction between the drug and the other excipients despite the high temperature during the melt granulation process.

## Conclusion

MTH floating tablets were developed with the combination of AG–HPMC, by thermoplastic granulation using stearic acid, according to an effervescent approach (NaCO_3_). Compared to AG as a polymer matrix, the AG–HPMC combination has improved the biopharmaceutical properties. The *in vitro* evaluation showed that the combined effect (AG–HPMC) resulted in the development of floating matrices with significantly reduced flotation times, 6 to 8 minutes for Fc3 (AG–HPMC k15M) and Fc6 (AG–HPMC k100M) respectively, instead of more than 1 hour for F1 (AG). Also, dissolution kinetics were slowed and extended, >8 hours for Fc3 and Fc6, instead of 2 hours for F1 (AG), with significantly reduced drug release rates, of approximately 95% and 91% for Fc3 and Fc6 respectively. The authors have demonstrated that these performances were dependent on the ratio of the combination (AG–HPMC). On the other hand, no significant influence of HPMC viscosity grade on the release kinetics of MTH has been revealed. The Fc3 and Fc6 formulations based on the AG–HPMC combination, at ratio, 1:3, appear to be the high-performances. Finally, based on the promising results of this study, it can be concluded that the AG–HPMC combination (1:3) can be considered as an interesting alternative to improve the flotation properties of the MTH tablets and to prolong the drug release.

## Ethical Issues

Not applicable.

## Conflict of Interest

The authors report no conflicts of interest. The authors alone are responsible for the content and writing of this article.

## Acknowledgments

The authors would like to thank the pharmaceutical laboratories El-Kendi, the Saïdal Annaba production unit, Inpha-Médis and Biocare for providing free raw materials. We also express our gratitude to the staff of the Saïdal Research and Development Center for their assistance with the study.
